# Batch crystallization of rhodopsin for structural dynamics using an X-ray free-electron laser

**DOI:** 10.1107/S2053230X15009966

**Published:** 2015-06-27

**Authors:** Wenting Wu, Przemyslaw Nogly, Jan Rheinberger, Leonhard M. Kick, Cornelius Gati, Garrett Nelson, Xavier Deupi, Jörg Standfuss, Gebhard Schertler, Valérie Panneels

**Affiliations:** a Laboratory for Biomolecular Research, Paul Scherrer Institute, OFLC/103, 5232 Villigen-PSI, Switzerland

**Keywords:** batch crystallization, GPCR, serial crystallography, FEL, dynamics

## Abstract

Rhodopsin is a membrane protein from the G protein-coupled receptor family. Together with its ligand retinal, it forms the visual pigment responsible for night vision. In order to perform ultrafast dynamics studies, a time-resolved serial femtosecond crystallography method is required owing to the nonreversible activation of rhodopsin. In such an approach, microcrystals in suspension are delivered into the X-ray pulses of an X-ray free-electron laser (XFEL) after a precise photoactivation delay. Here, a millilitre batch production of high-density microcrystals was developed by four methodical conversion steps starting from known vapour-diffusion crystallization protocols: (i) screening the low-salt crystallization conditions preferred for serial crystallography by vapour diffusion, (ii) optimization of batch crystallization, (iii) testing the crystal size and quality using second-harmonic generation (SHG) imaging and X-ray powder diffraction and (iv) production of millilitres of rhodopsin crystal suspension in batches for serial crystallography tests; these crystals diffracted at an XFEL at the Linac Coherent Light Source using a liquid-jet setup.

## Abstract 2

A new batch preparation method is presented for high-density micrometre-sized crystals of the G protein-coupled receptor rhodopsin for use in time-resolved serial femtosecond crystallography at an X-ray free-electron laser using a liquid jet.

## 1. Introduction   

Structure determination of membrane proteins has always been challenging owing to, among other factors, low expression levels and protein stability (Bill *et al.*, 2011 ▸). Despite the development of several novel methods for stabilization and crystallization (Tate, 2012 ▸; Rummel *et al.*, 1998 ▸; Liu *et al.*, 2014 ▸), membrane proteins, particularly G protein-coupled receptors (GPCRs), commonly yield crystals of insufficient size for classical crystallography (Smith *et al.*, 2012 ▸).

A promising new approach for the structure determination (Boutet *et al.*, 2012 ▸) of difficult-to-crystallize membrane proteins (Liu *et al.*, 2014 ▸), as well as for protein dynamics studies (Spence *et al.*, 2012 ▸; Neutze & Moffat, 2012 ▸; Tenboer *et al.*, 2014 ▸), arises from the advent of X-ray free-electron lasers (XFELs) as radiation sources (Gaffney & Chapman, 2007 ▸). XFELs can operate in a ‘diffraction-before-destruction’ regime (Neutze *et al.*, 2000 ▸) and allow the collection of data from many micrometre-sized crystals each generating a partial snapshot of reciprocal space (Chapman *et al.*, 2011 ▸; Barty *et al.*, 2012 ▸).

In order to decipher the ultrafast structural changes in the GPCR rhodopsin upon photoactivation, we designed a pump–probe experiment at an XFEL using a time-resolved serial femtosecond crystallography (SFX; Chapman *et al.*, 2011 ▸) setup. Micrometre-sized to submicrometre-sized crystals in a high-density suspension are delivered using a gas-focused liquid jet (DePonte *et al.*, 2008 ▸; Weierstall *et al.*, 2012 ▸), photoactivated by a pump laser and probed with the XFEL after a precise time delay (Aquila *et al.*, 2012 ▸). The success of a SFX experiment is highly dependent on crystal quality, size, density and quantity. However, all previously described crystallization conditions for wild-type dark-state rhodopsin (Palczewski *et al.*, 2000 ▸; Li *et al.*, 2004 ▸; Okada *et al.*, 2004 ▸; Salom *et al.*, 2006 ▸) are not suited for time-resolved SFX, since they contain high concentrations of salt and yield only a few large crystals (50–150 µm) in small-scale vapour-diffusion setups. Here, we describe the batch crystallization of rhodopsin, leading to a dense suspension of microcrystals under conditions suitable for SFX.

## 2. Materials and methods   

### 2.1. Materials   

Dark-adapted frozen bovine retinae were purchased from J. A. & W. L. Lawson Co., Lincoln, Nebraska, USA. The detergents tetraethylene glycol monooctyl ether (C_8_E_4_) and *N*,*N*-dimethyldodecylamine *N*-oxide (LDAO; 30% solution) were obtained from Bachem Ltd and Sigma–Aldrich, respectively. Concanavalin A Sepharose 4B and Q Sepharose Fast Flow resins were both obtained from GE Healthcare. MRC2 crystallization plates were obatined from SWISSCI AG. The MicroRT powder diffraction screening kits were obtained from MiTeGen. All work involving rhodopsin was carried out under dim red light.

### 2.2. Rhodopsin purification   

ROS membranes were isolated from bovine (*Bos taurus*) retinae and wild-type rhodopsin was purified as described in a previous publication (Edwards *et al.*, 2004 ▸) with the following modifications: (i) the first concanavalin A affinity chromatography step was scaled up three times and (ii) the Mono Q column was replaced by a Q Sepharose Fast Flow column using the same protocol as in Edwards *et al.* (2004 ▸) [with a buffer composition of 0.2%(*w*/*v*) detergent C_8_E_4_, 20 m*M* Tris pH 8.5, 1 m*M* Na_2_EDTA, 2 m*M* MgCl_2_, 1 m*M* β-mercapto­ethanol] but using the cOmplete protease-inhibitor cocktail from Roche and a reduced flow of 1 ml min^−1^. The fractions containing rhodopsin were concentrated to 11–15 mg ml^−1^ using an Amicon Ultra centrifugal filter with an Ultracel-30 membrane (Merck Millipore). Approximately 15–20 mg of pure rhodopsin was obtained from 100 retinae.

### 2.3. Rhodopsin crystallization   

#### 2.3.1. Rhodopsin crystallization by vapour diffusion   

Rhodopsin at 11–15 mg ml^−1^ was mixed in dim-light conditions with the same volume (200 nl) of precipitants from various commercial screens using a Mosquito nanolitre liquid-handling crystallization robot from TTP Labtech and the mixture was equilibrated against 50 µl reservoir solution in a MRC2 crystallization plate.

#### 2.3.2. Batch crystallization of rhodopsin   

**Figure 1:**
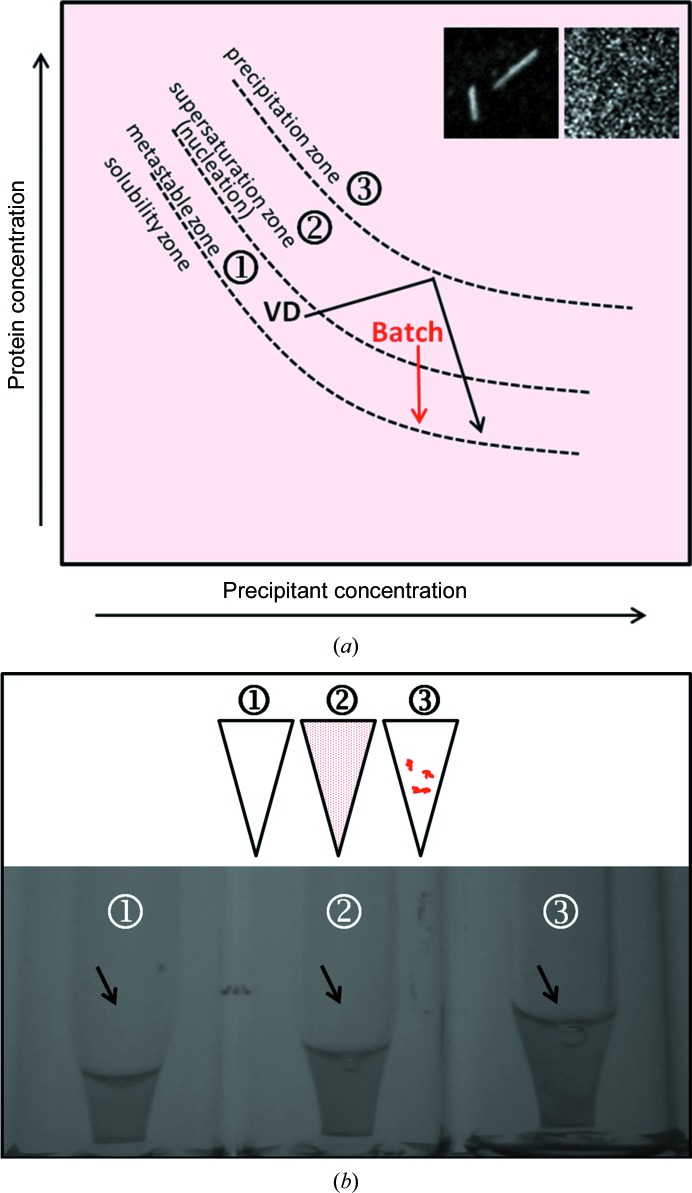
Batch crystallization of rhodopsin. (*a*) Phase diagram representing the changes made to transform vapour-diffusion conditions to batch crystallization in order to obtain higher nucleation and micrometre-sized crystals of rhodopsin for SFX. The arrows in black and red show the routes to reach the nucleation and metastable zones during the crystallization process for the vapour-diffusion (VD) and batch methods, respectively. The insets show SHG images of rhodopsin crystals grown by vapour diffusion (left; long needles) and by batch (right; grain-shaped micrometre-sized crystals) (both insets represent a 200 µm square). The circled numbers refer to the phases ‘metastable zone’ (1), ‘supersaturation zone’ (2) and ‘precipitation zone’ (3), which are successively reached when increasing the precipitant and/or protein concentration. (*b*) Three batch titrations of the precipitant into the rhodopsin solution under dim-light conditions. The upper and lower panels each show a scheme and a picture of an aliquot of an incomplete titration (tube 1; 20 µl rhodopsin + 32 µl precipitant) until low supersaturation [see label 1 in the phase diagram in (*a*)], an ideal batch titration (tube 2; 20 µl rhodopsin + 40 µl precipitant) until moderate supersaturation [see label 2 in the phase diagram in (*a*)] or an overtitration (tube 3; 20 µl rhodopsin + 50 µl precipitant) with an excess of precipitant inducing irreversible precipitation [see label 3 in the phase diagram in ( ▸ *a*)].

Batch crystallizations were set up by rapidly mixing the precipitant into the concentrated pure rhodopsin into a vial with a micro-insert (8004-HP-H/i3µ, Infochroma AG; Fig. 1 ▸ *b* and Table 1 ▸) and were incubated in the dark at 18°C. The titration experiments were conducted by mixing one volume of precipitant (*i.e.* 20 µl) into one volume of rhodopsin (20 µl) and titrating further with several small volumes (*n* × 2 µl) until the solution turned opalescent.

### 2.4. Crystal characterization   

#### 2.4.1. SHG imaging of rhodopsin crystals   

The crystallinity of the different batch suspensions was assessed using the second-harmonic generation (SHG) technology (Kissick *et al.*, 2011 ▸) in parallel with Ultraviolet Two-Photon Excited Fluorescence (UV-TPEF) imaging using a SONICC (second-order nonlinear imaging of chiral crystals) imager (Formulatrix; Wampler *et al.*, 2008 ▸). While UV-TPEF imaging allows the detection of UV fluorescence and is therefore most likely to be indicative of proteinaceous material, SHG imaging measures the crystallinity of the sample. It should be stressed that these methods are complementary and also that SHG imaging has its limitations: for instance, it can give false-negative results in rare instances when the crystal packing is of high symmetry and it can generate false-positive results if, for example, a salt crystal grows out the precipitant (Wampler *et al.*, 2008 ▸). Microlitre aliquots of the crystals grown in vials were transferred onto the drop wells of a MRC2 plate and signals were collected first at medium SHG power (350 mW laser power) and then at high SHG power (450 mW laser power) in order to detect the smallest possible crystals.

#### 2.4.2. Crystal density   

The crystal density was analyzed using a Neubauer cell chamber (NeoLab) and a MZ16 Leica stereomicroscope. The Neubauer chamber was filled with an aliquot of sample undiluted or diluted 10, 100 and 1000 times into the precipitant and the crystals were counted.

### 2.5. X-ray diffraction   

#### 2.5.1. Powder diffraction   

An aliquot of the crystal suspension, usually 30 µl, was pipetted into a MicroRT capillary (MiTeGen). The polyester capillary was then centrifuged in an Eppendorf tube at 500*g* for 5 min (Eppendorf Centrifuge 5424R) to concentrate the crystal suspension and mounted on a MiTeGen magnetic support for powder diffraction at the PXIII beamline of the Swiss Light Source (SLS), Villigen-PSI, Switzerland adapted for measurements in dim red light (5 keV, 10 s exposure, 100% transmission, 720° oscillation, 700 mm detector distance, one single image).

#### 2.5.2. X-ray diffraction at the LCLS   

Rhodopsin microcrystals (2–4 µm diameter) grown in batch suspension were loaded under dim light into the reservoir of a liquid jet (Weierstall *et al.*, 2012 ▸) and subjected in a serial manner to the 70 fs pulses of the XFEL (energy 6.7 keV) on the CXI (coherent X-ray imaging) beamline of the Linac Coherent Light Source (LCLS).

## 3. Results and discussion   

**Figure 2:**
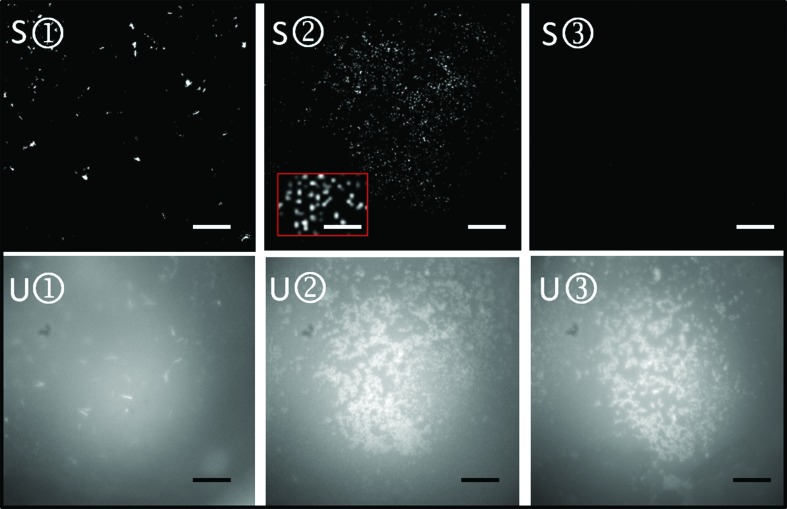
Selection of crystalline batch suspensions using SHG imaging microscopy. Rhodopsin batch crystallization trials [precipitant: 0.15 *M* dl-malic acid pH 7.0, 20%(*w*/*v*) PEG 3350] from the vials in Fig. 1 ▸(*b*) were tested for crystallinity by SHG imaging at 350 mW laser power (S1–S3) and for UV fluorescence by UV-TPEF (U1–U3). The scale bars represent 150 µm. The inset in the red box represents an enlargement of the typical grain-shaped micrometre-sized crystals (scale bar 50 µm) used in serial femtosecond crystallography.

**Figure 3:**
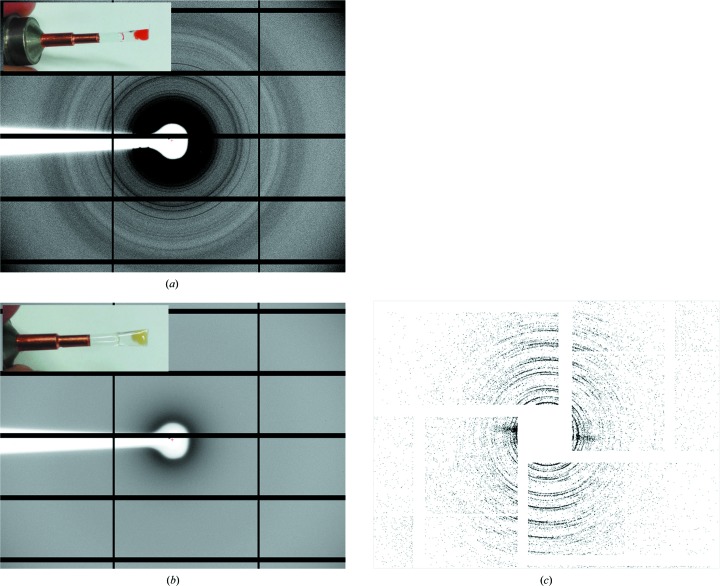
Diffraction tests of rhodopsin microcrystals at the SLS synchrotron and at the X-ray free-electron laser (XFEL). At the synchrotron, powder diffraction of a pellet of rhodopsin crystals kept at room temperature in the dark (*a*) or for 5 min in white light (*b*) was performed on the PXIII beamline of the SLS (5 keV, 10 s exposure). Diffraction rings are visible up to ∼9 Å resolution. After illumination, the pellet turned from red [inset in (*a*); retinal chromophore in the *cis* conformation] to yellow [inset in (*b*); retinal in the *trans* conformation], the crystals became disordered and the diffraction disappeared (*b*). At the XFEL, the rhodopsin crystals were tested on the CXI beamline of the LCLS at SLAC using a liquid jet. (*c*) shows a virtual powder diffraction pattern obtained by merging 2186 ‘hits’ extending to a resolution of 4–5 Å. This powder pattern shows an anisotropic distribution of Bragg peaks, which could reflect a preferred orientation of the crystals in the liquid stream.

Here, we describe the transformation of the rhodopsin crystallization condition leading from the single needles of approximately 150 µm (not shown; see Fig. 3 of Riekel *et al.*, 2005 ▸) obtained by Edwards *et al.* (2004 ▸) to conditions that result in a dense suspension of micrometre-sized crystals (Fig. 1 ▸ *a)*. These have been described to be ideal for SFX (Schlichting & Miao, 2012 ▸). In this study, wild-type rhodopsin from bovine retinae purified using a slightly modified version of the protocol of Edwards *et al.* (2004 ▸) was first subjected to several crystallization screens (Index HT and MembFac from Hampton Research and MemStart and MemSys HT-96 from Molecular Dimensions). This screening step was performed in order to find crystallization conditions without high salt concentrations, as strong diffraction from occasional salt crystals can damage the sensitive detectors used at XFELs (Carini *et al.*, 2014 ▸). Importantly, all crystallization and purification steps need to be performed under dim red light to prevent irreversible rhodopsin photoactivation. Condition H7 of the Index HT screen [0.15 *M* dl-malic acid pH 7.0, 20%(*w*/*v*) PEG 3350; Table 1 ▸] produced a shower of needles of 100 µm in length (data not shown) presenting the same type of packing (space group *P*6_4_, data not shown) as the crystals obtained at high salt concentrations by Edwards and coworkers. In order to increase the nucleation rate and decrease the crystal size, batch crystallization was performed by dropwise titration of the precipitant into the rhodopsin preparation until a moderate supersaturation state (Fig. 1 ▸ *a*, labelled 2) was reached, as judged by the presence of a stable pale opalescence (Fig. 1 ▸ *b*, tube 2). SHG imaging (Fig. 2 ▸) and powder diffraction at the SLS synchrotron (Fig. 3 ▸ *a*) were used to confirm the crystallinity of the grain-shaped microstructures from tube 2 (labelled S2 in Fig. 2 ▸). Both powder diffraction (Fig. 3 ▸ *b*) and SHG signals (not shown) typically disappeared upon exposure to white light owing to rhodopsin activation followed by crystal disordering. Interestingly, the batch prepared in the metastable zone (Fig. 1 ▸ *a*, labelled 1) showed a clear protein solution (Fig. 1 ▸ *b*, tube 1), but a few long rhodopsin needles were observed by UV-TPEF and SHG imaging (U1 and S1 in Fig. 2 ▸).

Batch crystallization was scaled up, and rhodopsin crystals tested by SFX at a density of 10^9^ crystals ml^−1^ showed initial X-ray diffraction patterns to 4–5 Å resolution at the XFEL at the LCLS, SLAC, Stanford, USA. The virtual powder pattern (Fig. 3 ▸ *c*) shows anisotropic distribution of Bragg peaks, which could reflect a preferred orientation of the grain-shaped crystals in the liquid stream or anisotropy of the microcrystals. Improvement of the crystal quality is in progress by using additives and changing the growth kinetics (McPherson & Gavira, 2014 ▸) using lower temperatures or methods such as free-interface diffusion, which has successfully been used for nanocrystallization of the PSII complex (Kupitz *et al.*, 2014 ▸).

## Figures and Tables

**Table 1:** Rhodopsin crystallization

Method	VD crystallization	Batch crystallization
Support	VDX† or MRC2 plates	Micro-insert tube
Temperature (K)	291	291
Final protein concentration (mgml^1^)	1115	1115
Detergent	C_8_E_4_	C_8_E_4_
Composition of precipitant	1*M* Li_2_SO_4_, 1.6% PEG 8000, 20%(*v*/*v*) glycerol	0.15*M* DL-malic acid pH 7.0, 20%(*w*/*v*) PEG 3350
Volume and ratio of drop	4 (2 + 2) l† or 400 (200 + 200) nl	300 (100 + 200) l
Volume of reservoir (l)	500† or 50	

† Edwards *et al.* (2004 ▸).
